# The Effects of Carnosine on Cognitive Function and Mental Health—A Systematic Review and Meta-Analysis

**DOI:** 10.3390/nu18091385

**Published:** 2026-04-28

**Authors:** Yung-Fang Hsiao, Zhongqi Fan, Yueh-Yin Fan, Mei Chung

**Affiliations:** 1 Friedman School of Nutrition Science and Policy, Tufts University, Boston, MA 02111, USA; 2 Department of Nutrition, Chung Shan Medical University, Taichung 40201, Taiwan; 3 Nutrition Evidence Synthesis and Data Analysis LLC, Boston, MA 02135, USA

**Keywords:** L-carnosine, zinc–L-carnosine, cognitive function, depression, mood, anxiety, quality of life

## Abstract

**Introduction:** Previous research has shown that L-carnosine (β-alanyl-L-histidine) can reduce cognitive decline and improve mental health outcomes, but an updated systematic review of the effects of carnosine alone or in combination with other supplemental nutrients or bioactive compounds on these interconnected outcomes is lacking. **Methods:** We searched multiple databases from 1 January 2006 to 30 June 2025 for clinical trials evaluating the effects of all forms of carnosine (e.g., L-carnosine, zinc–L-carnosine) alone or in combination with other supplements on cognition, brain structure and function, mood, depression, or quality of life (QOL) outcomes. The Cochrane Risk of Bias (ROB) 2.0 tool was used to assess the ROB in randomized controlled trials (RCTs). When data were sufficient, random-effects meta-analyses were conducted. Strength of evidence (SoE) across studies was rated using the GRADE approach. **Results:** A total of 13 distinct studies (12 RCTs; 1 single-arm trial) involving healthy adults and patients with psychiatric or neurocognitive disorders were included. Studies were also heterogeneous in carnosine supplement dosage and duration. Overall 58% of included RCTs were rated ‘some concerns’ for ROB. Ten RCTs evaluated cognitive function, seven RCTs and one single-arm trial assessed mood and depression, four RCTs measured QOL, and three RCTs examined brain structure and function. Results from five RCTs found no significant differences in the majority of the cognitive function measures between L-carnosine supplement and placebo, but random-effects meta-analysis of three RCTs from a single research team found that anserine/L-carnosine supplementation significantly improved WMS-LM2 scores (pooled net change = 1.70; 95% CI 0.19, 3.2; *I*^2^ = 58.3%) but not WMS–Local Memory Immediate Recall (LM1) scores (pooled net change = 0.76; 95% CI −0.18, 1.71; *I*^2^ = 8.5%). Additionally, meta-analysis results showed that L-carnosine combined with anserine or antioxidant supplementation significantly improved the MMSE score compared to placebo (pooled net change = 0.62; 95% CI 0.23, 1.01), with small statistical heterogeneity (*I*^2^ = 21.3%). Most of the studies did not show significant effects in a wide range of mood and depression outcome measures or health-related QOL (data cannot be meta-analyzed). **Conclusions:** A low strength of evidence suggests that L-carnosine supplement combined with anserine or antioxidants can slow cognitive function decline among healthy elderly or patients with probable Alzheimer’s Disease or mild neurocognitive disorder. More high-quality RCTs are needed to verify these findings and to improve the certainty level of this body of evidence.

## 1. Introduction

L-carnosine (β-alanyl-L-histidine) is a naturally occurring bioactive dipeptide composed of β-alanine and L-histidine, predominantly found in tissues with high metabolic demands such as skeletal muscle and the central nervous system [1,2]. Mediated by carnosine synthase, the endogenous synthesis of L-carnosine is rate-limited by the availability of β-alanine [3,4]. Physiologically, L-carnosine functions as an intracellular pH buffer, antioxidant, and anti-inflammatory agent, and protects against protein glycation and mitochondrial dysfunction [1,4]. Age-associated declines in muscle carnosine content have been associated with reduced exercise capacity and increased fatigue, whereas reductions in brain carnosine levels have been linked to oxidative damage, protein aggregation, and cognitive impairment in neurodegenerative conditions or aging [5,6,7]. Given these diverse physiological roles, the potential health effects of L-carnosine are vast and heterogeneous.

To systematically map this extensive landscape and prioritize domains for quantitative synthesis, we first conducted a scoping review that searched across multiple databases (Supplementary Materials S1 and S2) to identify all peer-reviewed human intervention studies, published between 1 January 2006 and 30 June 2025, examining the effects of carnosine (β-alanyl-L-histidine or zinc–L-carnosine) alone or in combination with other supplemental nutrients or bioactive compounds on a broad variety of prespecified health outcomes of interest (Supplemental Table S1). Zinc–L-carnosine, also called polaprezinc, is a chelated compound that contains L-carnosine and zinc. The goal of this scoping review is to identify research areas for which sufficient evidence warrants a systematic review (SR) and meta-analysis. We found that research on the effects of L-carnosine on cognitive function, brain structure and function, mood and depression, and quality of life (QOL) outcomes has sharply increased in the last 10 years. A previous SR [8] identified five randomized controlled trials (RCTs), published up to March 2021, investigating the potential effects of L-carnosine on preventing cognitive decline and depressive symptoms in the elderly. This SR did not find significant effects on these outcomes in individual RCTs, but their meta-analysis of three RCTs showed a small effect (standardized mean difference: −0.25, 95% CI −0.46, −0.04) against cognitive decline, comparing L-carnosine interventions along with anserine to the controls [8]. Another recent SR and meta-analysis examined the effects of histidine-containing dipeptides (anserine/L-carnosine, L-carnosine, β-alanine) on psychological and mental health outcomes, and the findings showed that supplementation with histidine-containing dipeptides significantly reduced depression scores and increased QOL measures compared with placebo [9]. Anserine (β-alanyl-3-methyl-L-histidine) is a methylated derivative of L-carnosine. Unlike L-carnosine, which is rapidly hydrolyzed by serum carnosinase (CN1), anserine is resistant to this enzymatic breakdown, thereby exhibiting greater serum stability and potentially higher bioavailability while maintaining similar physiological functions [10]. Furthermore, β-alanine, the rate-limiting precursor for L-carnosine synthesis, is also considered an effective alternative supplementation strategy to increase endogenous carnosine levels [3].

Cognitive function and mental health are closely interconnected components of well-being in adults with cognitive impairment [11]. These factors collectively influence overall QOL, indicating that preserving cognitive function is essential for maintaining health [11]. Therefore, we decided to conduct an SR to synthesize all human trial evidence identified by our scoping review for the effects of carnosine (β-alanyl-L-histidine or zinc–L-carnosine) alone or in combination with other supplemental nutrients or bioactive compounds on these interconnected outcomes.

## 2. Materials and Methods

This SR was conducted following the Cochrane Handbook for Systematic Reviews of Interventions [12]. We reported findings in accordance with the 2020 Preferred Reporting Items for Systematic Reviews and Meta-Analyses (PRISMA) statement [13]. The literature searches and selection processes were performed for a broader scoping review (referred to as “the scoping review” herein) following prespecified scoping review study eligibility criteria (Supplementary Material S2). The scoping review protocol was registered on the Open Science Framework (OSF; https://doi.org/10.17605/OSF.IO/N28B4, accessed on 19 March 2026).

### 2.1. Data Sources and Searches

The search strategy was developed utilizing bibliographic databases, including PubMed^®^ (from 2006 to 26 June 2025), the Cochrane Central Register of Controlled Trials (from 2006 to 9 July 2025), and EMBASE (from 2006 to 9 July 2025). For the scoping review, our search strategies incorporated keywords related to all forms of carnosine (e.g., L-carnosine, zinc–L-carnosine) or carnosine-containing supplements, which were published in English, excluded any review articles, and employed indexing terms specific to each database (e.g., Medical Subject Headings [MeSH] for MEDLINE^®^ and Emtree terms for Embase) (Supplementary Material S1). In addition to searching bibliographic databases, we conducted reference mining within relevant previous narrative and systematic reviews to ensure no relevant studies were omitted.

### 2.2. Study Selection

Abstract screening, full-text review, and risk of bias (ROB) assessments were conducted using PICO Portal, a web-based literature review platform (PICO Portal, St. Petersburg, FL, USA), accessed on 1 July 2025. Abstract screening and full-text review were conducted by at least two independent investigators, with conflicts resolved through consensus. For the scoping review, studies that supplemented adult populations (aged 18 years or older without disease restrictions) with L-carnosine alone or in combination with other nutrients were deemed eligible for inclusion. Studies and research involving non-human subjects, as well as those involving infants, children, and adolescents, were excluded. Eligible study designs included all types of clinical trials, such as parallel or crossover RCTs, non-randomized controlled trials or crossover studies, and uncontrolled (single-arm) trials. Observational studies, reviews, SRs, case studies, or articles that were not peer-reviewed were excluded. To qualify for inclusion, study reports were required to quantify L-carnosine intake from supplements and assess relevant outcomes. We included studies examining various dosage levels and those comparing L-carnosine to a placebo, any other controls, or no comparator. Following full-text review, a decision was made to focus on outcomes related to cognition, brain structure or function, mood or depression, or QOL for the subsequent SR. The eligibility criteria for the present SR, which conformed to the Population Intervention/Exposure Comparator Outcome (PI/ECO) framework, are outlined in Table 1.

### 2.3. Data Extraction

Standardized data extraction forms were created to extract study and participant characteristics and quantitative results. All data were extracted by one investigator and checked by another investigator. The study characteristics data included publication year, country, design, and duration. Additionally, we extracted data regarding the type of L-carnosine, dosage, frequency, comparator type, and description, along with each study’s outcome measures related to cognitive function, brain structure, mood or depression, and QOL. Participant characteristics data comprised the enrolled or randomized sample size, the percentage of male participants, health status, and the mean baseline data for age within each study. Key findings of individual studies were summarized using predefined symbols defined in the footnotes of the result summary tables.

### 2.4. Risk of Bias

All included RCTs were independently evaluated for ROB by 2 investigators utilizing the Cochrane risk-of-bias tool for randomized trials (RoB-2) [14], with any discrepancies resolved through consensus. Original trial registrations or protocols of all included RCTs were sought for ROB assessments. RCTs were assessed on 21 items across 5 domains, which measure potential bias originating from the randomization process, intervention adherence, missing outcome data, outcome measurement, and the selection of reported results. Each item was rated with one of the following options: ‘yes’, ‘probably yes’, ‘probably no’, ‘no’, or ‘no information’. Subsequently, five domains were evaluated as either ‘Low ROB’, ‘Some concern’, or ‘High ROB’ based on the responses to the items, in accordance with the algorithm outlined in the Cochrane RoB-2 guidance. The Investigators determined the overall ROB as either ‘Low ROB’, ‘Some concern’, or ‘High ROB’ by evaluating ratings in each domain. For included single-arm trials, a default classification of ‘High ROB’ was applied due to the absence of control groups.

### 2.5. Qualitative Data Synthesis

Data were synthesized for each outcome separately. Summary tables were created to present key study features, ROB, and key findings to facilitate qualitative synthesis. Only a small number of studies provided sufficient data for meta-analyses; thus, meta-analysis results were compared with studies that were not meta-analyzed narratively. Strength of evidence (SoE) across studies was rated using the Grades of Recommendation, Assessment, Development, and Evaluation (GRADE) approach [15]. For each outcome, SoE profile tables were compiled to report the number and design of studies, as well as the overall limitations, imprecision, inconsistency, indirectness, and publication bias. Overall SoE (i.e., very low, low, moderate, or high) was graded by the consensus of the entire research team to indicate the degree of confidence that estimated effects from reviewed evidence were close to the true effect.

### 2.6. Meta-Analyses

Meta-analyses of RCTs were planned to combine the effects of L-carnosine interventions on cognition, brain structure and function, mood, depression, or QOL outcomes when 3 or more RCTs reported sufficient quantitative data for analysis. Quantitative data were extracted using a standardized Excel spreadsheet developed to facilitate pre-analysis calculations for missing data and downstream meta-analysis. Several publications of the same RCT conducted by the same group of investigators in Japan reported outcomes from overlapping study participants [16,17,18]. We carefully inspected the reported data to ensure that only one effect size from each publication was included in each meta-analysis. For effect size calculations, we extracted both baseline and endpoint results, and/or reported change statistics (i.e., mean change and variance of mean change from baseline within group).

Random-effects (DerSimonian-Laird method) meta-analyses were conducted to account for the heterogeneity across included studies. Prior to performing meta-analyses, reported within-group standard errors or 95% confidence intervals (CIs) were converted to standard deviations (SDs). When only repeated-measures data were reported, within-group change (mean change) was calculated as the difference between baseline and final mean measures. We imputed missing SDs of mean change using the reported baseline and final SDs and a common correlation coefficient of 0.9, based on correlations between baseline and final SDs from other studies included in the meta-analysis. Sensitivity analyses using alternative r values of 0.5 were performed. Finally, the net change (difference in the two within-group mean changes from baseline between the intervention and control groups) was calculated as a measure of effect for meta-analysis.

Meta-analysis results were considered statistically significant if the confidence intervals for pooled effect sizes excluded zero. Cochrane’s Q statistic was computed to quantify statistical heterogeneity, with a *p*-value of ≤0.1 regarded as statistically significant. *I*^2^ values of 25%, 50%, and 75% were interpreted as indicative of low, moderate, and high heterogeneity, respectively. All calculations and meta-analyses were performed using Stata SE software (version 18.0; Stata Corporation, College Station, TX, USA). Meta-analyses were performed using *metan*.

## 3. Results

**Figure 1:**
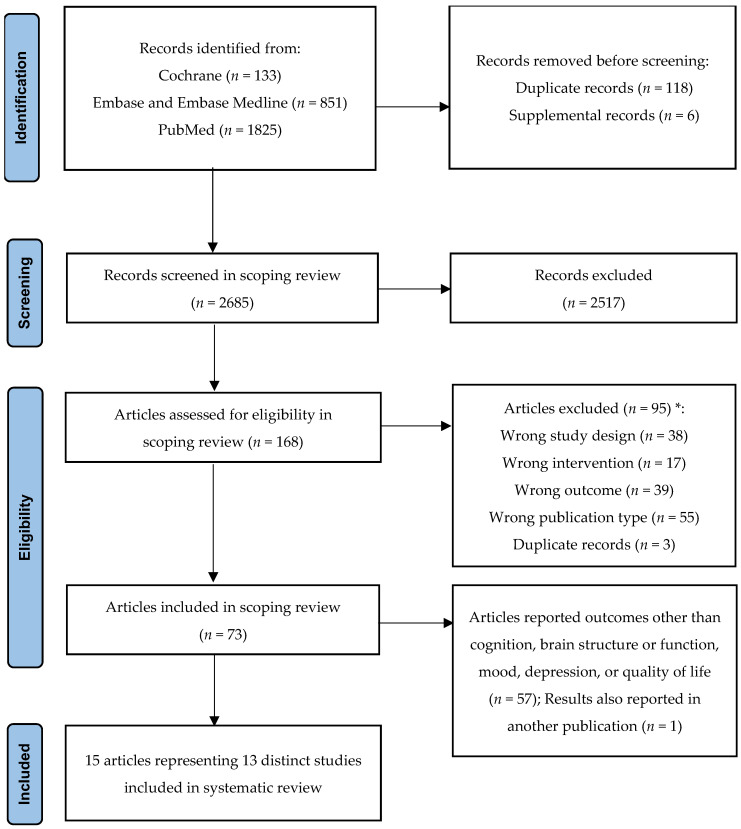
PRISMA flowchart of literature search and study selection. * Reports can be excluded for more than one reason.

After screening 2809 records, 73 articles met the inclusion criteria of the scoping review. For the present SR, articles reporting outcomes related to cognitive function, brain structure/function, mood, depression, or QOL were included. Thus, a total of 15 articles representing 13 distinct studies (12 RCTs; 1 single-arm trial) were included in this SR. The literature searches and selection process are summarized in Figure 1. Among the included 12 RCTs published in 14 publications (Table 2 and Table 3), six utilized a combination of L-carnosine and other nutrients (e.g., anserine) supplementations [10,16,17,18,19,20,21,22], while the other six trials administered L-carnosine supplementation alone [23,24,25,26,27,28]. Most RCTs were double-blind, placebo-controlled, parallel trial designs, except for one, which was an unblinded controlled trial [21]. The single-arm trial administered zinc–L-carnosine supplement (Table 4) [29]. Across all studies, administered L-carnosine dosages ranged from 100 to 2000 mg/day, with intervention durations spanning 6 weeks to 12 months. Sample sizes ranged from 29 to 299 participants (aged 18 to 94 years). Study populations were heterogeneous, including healthy adult individuals or elderly people [10,16,17,18,21,22,26] as well as those with psychiatric, neurocognitive, or metabolic conditions, including Gulf War illness [23], schizophrenia [24,27], major depressive disorder [28], Alzheimer’s disease [19], mild neurocognitive disorder [20], binge eating disorder or bulimia nervosa [29], and prediabetes or well-controlled type 2 diabetes [25]. Geographically, these studies were conducted in Japan (*n* = 5 studies), the United States (*n* = 4 studies), Australia (*n* = 1 study), India (*n* = 1 study), Iran (*n* = 1 study), and Poland (*n* = 1 study). Regarding outcome measures, 10 RCTs (published in 12 articles) evaluated cognitive function, seven RCTs (published in nine articles) and one single-arm trial assessed mood and depression, four RCTs (published in six articles) measured QOL, and three RCTs (published in four articles) examined brain structure and function (Table 3 and Table 4). Only 34% of the included RCTs reported adverse events, including constipation [24,27,28,29], drowsiness and dizziness [24,28,29], nausea [27,28,29], dry mouth [24,27], appetite change [24,28], elevation of liver enzymes [23,29], paresthesia [24], and stiffness [28]. Of these, the original authors reported no significant difference in the frequency of adverse effects between the intervention group and the control group [24,27,28] or reported that the adverse effects were not related to the intervention [23].

**Figure 2:**
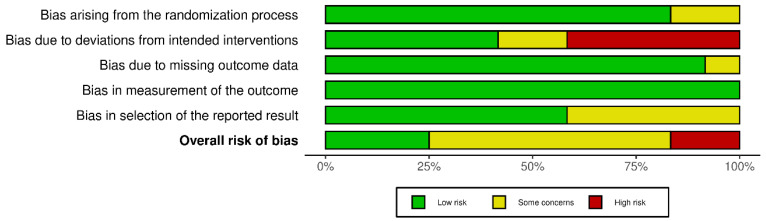
Domain-specific and overall risk-of-bias judgment results for 12 included randomized controlled trials.

Overall ROB of the 12 RCTs is predominantly characterized by some concerns (*n* = 7 studies), with a smaller proportion of studies exhibiting low (*n* = 3 studies) or high (*n* = 2 studies) ROB (Figure 2). ROB was primarily driven by deviations from intended interventions (e.g., non-compliance or dropouts) and by selective result reporting bias (Supplementary Figure S1). The single-arm trial was rated as ‘High ROB’ due to the absence of a control group.

### 3.1. Cognitive Function Outcomes

Five RCTs (one ‘low risk’; four ‘some concerns’) [23,24,25,26,27] evaluated the effects of L-carnosine supplement alone compared to placebo for 6 weeks to 6 months on a variety of cognitive function measures (Table 3). The L-carnosine supplement dosages and regimens also varied across these RCTs. These RCTs mostly found no significant differences in the cognitive function measures between L-carnosine supplementation and placebo. Only two RCTs (one rated ‘low risk’; one ‘some concern’) [23,24] reported that L-carnosine supplementation significantly improved some cognitive function scores (i.e., Wechsler Adult Intelligence Scale Digit Symbol Substitution Test, Set Shifting test, and Strategic Target Detection) compared to placebo.

**Figure 3:**
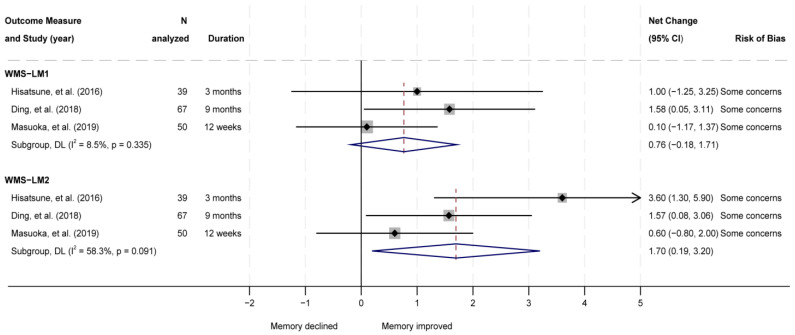
Random-effect meta-analysis of randomized controlled trials assessing WMS-LM1 and WMS-LM2 outcomes comparing supplementation with anserine and carnosine (3:1) to placebo [10,16,20]. Box size indicates study weight. WMS-LM1 = Wechsler Memory Scale–Local Memory Immediate Recall; WMS-LM2 = Wechsler Memory Scale–Local Memory Delay Recall.

**Figure 4:**
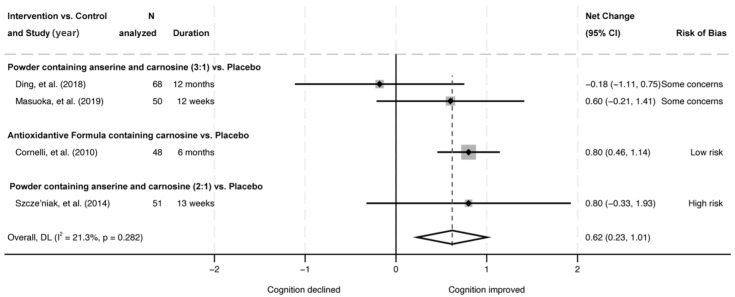
Random-effects model meta-analysis of randomized controlled trials measuring MMSE in participants given dietary supplements containing carnosine [10,19,20,22]. Box sizes represent study weight. MMSE, Mini–Mental State Examination.

Another five RCTs (one ‘low risk’; three ‘some concerns’; one ‘high risk’), published in seven articles [10,16,17,18,19,20,22] compared the effects of L-carnosine supplement combined with anserine or other antioxidants to placebo (Table 3 and Table 4). Among these, three RCTs were conducted in the same metro area of Japan using the same intervention (1 g of anserine/L-carnosine 3:1 ratio per day) compared to placebo among healthy elderly or patients with mild neurocognitive disorder [10,16,17,18,20]. All three RCTs did not show significant differences in Alzheimer’s Disease Assessment Scale–Cognitive Subscale between groups, but all showed improvements in Wechsler Memory Scale (WMS)–Local Memory Delay Recall (WMS-LM2) scores. Random-effect meta-analysis combined with the results of WMS scores showed that anserine/L-carnosine supplementation significantly improved WMS-LM2 scores (pooled net change = 1.70; 95% CI 0.19, 3.2) but not WMS–Local Memory Immediate Recall (LM1) scores (pooled net change = 0.76; 95% CI −0.18, 1.71) compared to placebo. The statistical heterogeneity was moderate (*I*^2^ = 58.3, *p* = 0.091) for the WMS-LM2 score and small (*I*^2^ = 8.5%, *p* = 0.335) for the WMS-LM1 score (Figure 3). Sensitivity analysis showed slightly more significant results (Supplementary Figure S2).Additionally, four of the five RCTs (one ‘low risk’; two ‘some concerns’; one ‘high risk’) [10,19,20,22] investigated the effects of L-carnosine supplement combined with anserine or other antioxidants on Mini–Mental State Examination (MMSE) outcome. The random-effects meta-analysis showed the MMSE score was significantly improved compared to placebo (pooled net change = 0.62; 95% CI 0.23, 1.01), with small statistical heterogeneity (*I*^2^ = 21.3%, *p* = 0.282) (Figure 4). Sensitivity analysis showed less significant results (Supplementary Figure S3). Two RCTs [20,22] also reported cognitive outcomes measured by other cognitive function scores, such as the Clinical Dementia Rating (CDR) and Short Test of Mental Status (STMS). Results were mostly null, except that the change score in global CDR was significantly greater for patients with mild neurocognitive disorder who were supplemented with 1 g of anserine/L-carnosine 3:1 ratio per day than those who received a placebo in 1 RCT [20].

Overall SoE was rated as ‘low’ for cognitive function outcomes across the 10 RCTs (Supplementary Table S2).

### 3.2. Brain Structure and Function Outcomes

Three RCTs (all rated ‘some concerns’) examined the changes in brain structure and function as intermediate outcomes of cognitive function, using functional Magnetic Resonance Imaging (MRI) or quantitative Electroencephalogram (EEG) spectrum intensity measures [10,16,18,20]. All three RCTs (published in four articles) were conducted in the same metro area of Japan using the same intervention (1 g of anserine/L-carnosine 3:1 ratio per day) compared to placebo among healthy elderly people or patients with mild neurocognitive disorder, and reported some significant changes in brain structure and function outcomes after anserine/L-carnosine supplementation compared to placebo (Table 2 and Table 3). Overall SoE was rated as ‘very low’ for brain structure and function outcome (Supplementary Table S2). 

### 3.3. Mood and Depression Outcomes

A wide range of mood and depression outcome measures were examined in seven RCTs (published in nine articles) (Table 2 and Table 3) [10,16,17,18,20,21,24,28] and one single-arm trial (Table 4) [29]. Of the seven RCTs, two RCTs (both rated ‘low risk’) examined the effects of L-carnosine supplement alone compared to placebo [24,28]. One RCT found that patients with major depressive disorder who received 400 mg twice daily L-carnosine supplementation for 6 weeks had significantly greater improvement in Hamilton Depression Rating Scale (mean difference = 3.15; 95% CI 0.45–5.84; *p* = 0.02) than those who received a placebo in addition to citalopram therapy [28]. However, the other RCT that investigated L-carnosine (with escalating dosing) as an adjunct therapy for 3 months did not find a significant difference in the Calgary Depression Scale for Schizophrenia (as one of the secondary outcomes) between patients with schizophrenia or schizoaffective disorder who received L-carnosine supplement than those who received placebo [24].

All five RCTs (one ‘low risk’; three ‘some concerns’; one ‘high risk’), comparing the effects of L-carnosine supplement combined with anserine or antioxidants to placebo, also investigated mood and depression outcomes (Table 2 and Table 3) [10,16,17,18,19,20,21,22]. Four of the five RCTs did not find significant differences in depression symptom scores as measured by the Beck Depression Inventory (BDI) or the Geriatric Depression Scale (GDS) between groups (Table 3) [10,16,17,18,19,20,22]. The other RCT (rated ‘high risk’) showed that cognitive behavioral therapy (CBT) with one bottle of the supplement soft drink (100 mL) containing 200 mg/d L-carnosine significantly improved Profile of Mood States (POMS) Tension–Anxiety and Fatigue scores but not POMS Depression score, compared to CBT therapy alone or in the absence of a treatment control group [21].

Lastly, the single-arm trial (rated ‘high risk’) examined the effects of zinc–L-carnosine complex in patients with binge eating disorder (BED; *n* = 22) or bulimia nervosa (BN; *n* = 7) receiving antidepressants. The study found that Quick Inventory of Depressive Symptomatology scores were steadily decreased for both BED and BN patients throughout the 1 week of therapy (Table 4) [29].

Overall SoE was rated as ‘low’ for mood and depression outcomes across the seven RCTs and one single-arm trial (Supplementary Table S2).

### 3.4. Quality of Life (QOL) Outcomes

Four RCTs (one ‘low risk’; four ‘some concerns’) measured health-related QOL using The 36-Item Short Form Health Survey (SF-36) comparing L-carnosine supplementation alone to placebo [23,24] or comparing anserine/L-carnosine (3:1 ratio) supplementation to placebo [10,16,17]. None of these RCTs showed a significant difference in SF-36 score between groups (Table 2 and Table 3). One RCT evaluating the Quality of Life Interview score (QOLI) found no significant difference between L-carnosine supplementation alone and placebo. Overall SoE was rated as ‘very low’ for QOL outcome (Supplementary Table S2).

## 4. Discussion

Current evidence from human RCTs suggests that L-carnosine combined with anserine or antioxidant supplementation can slow cognitive function decline among healthy elderly or patients with probable Alzheimer’s Disease or mild neurocognitive disorder. These effects are supported by the favorable changes in brain structure and function outcomes as measured by functional MRI or EEG spectrum intensity measures in those subjects who received L-carnosine combined with anserine supplementation. On the contrary, the effects on cognitive function outcomes are mostly null in RCTs comparing L-carnosine supplement alone to placebo. Evidence from highly heterogeneous RCTs among various patient populations suggests mixed effects on mood and depression outcomes, and null effects on health-related QOL comparing L-carnosine alone or in combination with other nutrients or bioactive compounds to controls. However, the strength of evidence was rated ‘low’ or ‘very low’, indicating low certainty in these effect estimates, and future research will likely change these conclusions.

The potential beneficial effects of L-carnosine on cognitive function are thought to be mediated through multiple neuroprotective mechanisms that counteract aging-related decline. It acts as an antioxidant by scavenging reactive species and upregulating the Nrf2 pathway [4,5], while concurrently mitigating neuroinflammation by suppressing TNF-α [8,9]. Beyond preserving cognitive capabilities, these anti-inflammatory properties, in conjunction with carnosine’s capacity to modulate glutamatergic neurotransmission, have been associated with improvements in mood and reductions in depressive symptoms [9,28]. Furthermore, carnosine inhibits neurotoxicity by preventing the aggregation of amyloid-beta (Aβ) and advanced glycation end-products (AGEs) [5,30], and maintains executive function by ensuring cerebral perfusion within the prefrontal cortex [10].

Dietary L-carnosine is absorbed and metabolized in the human intestine. Upon intestinal absorption through the peptide transporter 1 (PEPT1), L-carnosine is rapidly hydrolyzed in the bloodstream by serum carnosinase (CN1) into β-alanine and L-histidine [3,30,31]. Thus, inter-individual variability in CN1 activity, influenced by carnosine dipeptidase 1 (CNDP1) gene polymorphisms, may further contribute to the heterogeneous clinical outcomes observed [3]. In contrast to L-carnosine, β-alanine bypasses serum degradation and is efficiently transported into neurons. However, individuals who received high-dose β-alanine supplementation have reported symptoms of paresthesia, which is an unpleasant sensation of tingling or itching caused by rapid absorption and high plasma concentrations, calling for a new formulation of β-alanine supplements [32]. Since β-alanine acts as the rate-limiting substrate for carnosine synthase, it thereby promotes the effective re-synthesis of carnosine stores [3,4,33]. Recent studies confirm that β-alanine supplementation elevates tissue carnosine levels and stimulates brain-derived neurotrophic factor (BDNF) [33], therefore preserving hippocampal structural integrity and enhancing cognitive performance in older adults [34]. Enhancing the bioavailability of carnosine through supplementation with β-alanine or in combination with anserine or other ingredients may offer greater clinical benefits across cognitive function, mental health, and QOL than carnosine supplementation alone.

The present SR provides updated evidence from a previous SR investigating the potential effects of L-carnosine on preventing cognitive decline and depressive symptoms in elderly people [8]. Our findings are consistent with the earlier SR, but unlike previous SRs, we synthesized the effects of L-carnosine alone and its combined effects with other supplemental nutrients or bioactive compounds separately to isolate the independent effect of L-carnosine. We conducted meta-analyses on three cognitive function outcomes—WMS-LM1, WMS-LM2, and MMSE—using their original units (as opposed to standardized effect size in the earlier SR) to enhance clinical interpretations of these results. Nevertheless, there are some limitations in the present SR, primarily stemming from the clinical and methodological heterogeneity across included studies. Specifically, the included trials covered a wide range of countries, age groups, and clinical conditions, resulting in substantial population heterogeneity. This limits how broadly the findings can be applied and makes comparisons across studies difficult. In addition, many trials had small sample sizes—some with as few as 29 participants—which increases the likelihood that results were influenced by random error. For example, cognitive, mood, and QOL outcomes were measured using multiple instruments, some of which require interviewer-administered and scored assessments. Because these processes can vary across assessors, there is a risk of inter-rater variability and human errors, which can contribute to random measurement errors. Although MMSE scores showed statistically significant improvement with carnosine supplementation, the net change was modest and may not be clinically meaningful, as it was below the commonly accepted minimal clinically important difference of 1–3 points [35]. Sensitivity analysis also showed that our meta-analysis of the MMSE outcome was not robust due to missing data. To avoid overinterpretation, we assessed the certainty of evidence using the GRADE approach rather than drawing conclusions based on a single measure of cognitive function. Regarding potential biases in the included studies, while most RCTs showed ‘some concerns’ in the ROB assessment, and two studies were rated as high ROB due to baseline differences in outcome parameters between groups (indicating unsuccessful randomization) and unblinded study design. Additionally, many RCTs performed multiple testing across many outcome measures, which increased the chance of false positive findings. Finally, because this SR only included English-language, peer-reviewed intervention studies, publication bias cannot be ruled out.

To enhance the body of evidence on and our understanding of the effects of L-carnosine on cognitive function and mental health, future RCTs should investigate the minimal effective dosage of L-carnosine and compare the effects of L-carnosine supplementation alone with that in combination with other nutrients or bioactive compounds. All high-quality RCTs should be sufficiently powered and utilize a double-blind design to minimize biases. Given the high burden of cognitive decline and mental health disorders, future prospective studies should investigate the long-term effects of early exposure to carnosine supplementation in the prevention of these clinical outcomes.

## 5. Conclusions

A low strength of evidence suggests that L-carnosine supplementation combined with anserine or antioxidants can slow cognitive function decline among healthy elderly or patients with probable Alzheimer’s Disease or mild neurocognitive disorder. More high-quality RCTs are needed to verify these findings and to improve the certainty level of this body of evidence.

## Figures and Tables

**Table 1:** Study eligibility criteria.

Category	Inclusion Criteria	Exclusion Criteria
Study designs	Any intervention study: Randomized controlled trials Non-randomized controlled trials Randomized or non-randomized cross-over trials Single-arm trials	Case-control studies Cohort studies Cross-sectional studies Narrative reviews Systematic reviews and meta-analysis Protocols Conference proceedings Abstracts Letters to the editor Case studies or case series In vitro and animal studies
Populations	Human subjects without age, sex, and disease restrictions	Non-human subjects
Interventions or exposures	Oral intake of L-carnosine (β-alanyl-L-histidine) or zinc–L-carnosine alone or combined with other supplements	Interventions are not administered orally (for example, eyedrops, skin cream or topical products)
Comparators	Placebo or other supplements	None
Outcomes	Cognition Cognitive function ^1^ Dementia Alzheimer’s disease Cognitive impairment Brain structure and function (intermediate outcome of cognition) Mood and depression Quality of life	Outcomes beyond cognition, brain structure and function, mood and depression, and quality of life

^1^ Cognitive function, as assessed by the following domains: global cognition, executive function, learning and memory, attention and processing speed, abstract reasoning and decision making, language function, and visuospatial and socio-motor function.

**Table 2:** Characteristics of randomized controlled trials.

Author, Year (Reference)	Country	Funding Source	N	Design	^1,2^ Mean Age [Range], Years	^2^ % Male	Health Status	Carnosine Intervention and Dosage	Comparator	Intervention Duration
**L-carnosine**										
Araminia et al., 2020 [28]	Iran	Government	58	Parallel RCT	~33.4 [18–60]	55.8%	Patients with major depression disorder	400 mg/d twice daily	Placebo	6 weeks
Baraniuk et al., 2013 [23]	USA	Government	34	Parallel RCT	~49.4 * [NR]	68%	Gulf War illness subjects	500 mg/d for 4 weeks, increased to 1000 mg/d for 4 weeks, and increased to 1500 mg/d for 4 weeks. After 12 weeks, 1500 mg/d was tapered by 500 mg per week until discontinued	Placebo	14 weeks
Chengappa et al., 2012 [24]	USA	Nonprofit and industry	70	Parallel RCT	~46.5 [18–65]	63%	Patients with schizophrenia or schizoaffective disorder	500 mg/d for a week, increased to 1000 mg/d for a week, increased to 1500 mg/d for a week, and increased to 2000 mg/d for 9 weeks	Placebo	3 months
Hariharan et al., 2025 [25]	Australia	Government	49	Parallel RCT	~51.2 [28–60]	69% *	Patients with prediabetes or early-stage well-controlled type 2 diabetes mellitus	2 g/d	Placebo	14 weeks
O’Toole et al., 2025 [26]	USA	Government	299	Parallel RCT	45 [NR]	42.1%	Healthy population	2 g/d	Placebo	12 weeks
Tharoor et al., 2023 [27]	India	Industry	100	Parallel RCT	NR [18–45]	66%	Patients with schizophrenia	400 mg/d for 3 months, and increased to 800 mg/d for 3 months	Placebo	6 months
**L-carnosine with other nutrients or bioactive compounds**										
Cornelli et al., 2010 [19]	USA	NR	52	Parallel RCT	~74.5 [NR]	39.6%	Patients with probable Alzheimer’s disease	Formula F (100 mg carnosine and antioxidant compounds: vitamins B, vitamin C and E, coenzyme Q10, beta-carotene, selenium, l-cysteine, Ginkobiloba)/d	Placebo	6 months
Ding et al., 2018 [10]	Japan	Government	84	Parallel RCT	~71.6 * [60–80]	42.6% *	Healthy population	1 g of anserine/carnosine (3:1 ratio)/d	Placebo	12 months
^3^ Hisatsune et al., 2016 [16]	Japan	Government and industry	39	Parallel RCT	~69.2 [60–78]	43.6%	Healthy population	1 g of anserine/carnosine (3:1 ratio)/d	Placebo	3 months
^3^ Katakura et al., 2017 [17]			60		~62.9 [41–78]	33.3%				
^3^ Rokicki et al., 2015 [18]			31		~63.6 [42–78]	32.2%				
Masuoka et al., 2019 [20]	Japan	Government	54	Parallel RCT	72.8 [49–86]	48% *	Patients with mild neurocognitive disorder	1 g of anserine/carnosine (3:1 ratio)/d	Placebo	12 weeks
Shirotsuki et al., 2017 [21]	Japan	Industry	87	Parallel RCT	~37.2 * [NR]	68.1% *	Healthy population	CBT with one bottle of the supplement soft drink (100 mL) containing 200 mg/d L-carnosine. The drink also contained 0.2 g protein, 18 g carbohydrate, and 40 mg sodium	CBT or control (no intervention)	6 weeks
Szcześniak et al., 2014 [22]	Poland	Government	56	Parallel RCT	80.8 * [65–94]	49% *	Nursing home residents with MMSE score > 15	1 g of anserine/carnosine (2:1 ratio)/d	Placebo	13 weeks

CBT, cognitive behavioral therapy; MMSE, Mini–Mental State Examination; NR, not reported; RCT, randomized controlled trials. ^1^ If the total mean (SD) was not reported for study participants, the table shows means as weighted averages (indicated by the “~” symbol) and includes separate group SDs reported in the original study (presented in parentheses, separated by a comma). ^2^ If the total mean (SD) was only reported for participants who finished the study, it is indicated with an *. ^3^ Participants in these articles originated from the same study conducted within the same recreated period.

**Table 3:** Key findings and overall risk of bias of randomized controlled trials.

Author, Year (Reference)	Outcome Analyzed	Key Findings ^1^	Overall ROB ^2^
**L-carnosine**			
Araminia et al., 2020 [28]	Mood and depression: HAM-D	HAM-D: ++	Low risk
Baraniuk et al., 2013 [23]	Cognition: TMT; WAIS-R Digit Symbol Quality of Life: SF-36	TMT: 0 WAIS-R Digit Symbol: ++ SF-36: 0	Some concerns
Chengappa et al., 2012 [24]	Cognition: SST; STDT; Auditory Digit Span; CPT; Working Memory; Word List Memory; Finger Tapping Speed Test Mood and depression: CDSS Quality of Life: QOLI, SF-36	SST: ++/0 STDT: ++ Auditory Digit Span; CPT; Working Memory; Word List Memory; Finger Tapping Speed Test: 0 CDSS: 0 QOLI: 0 SF-36: 0	Low risk
Hariharan et al., 2025 [25]	Cognition: TMT; DSST; Stroop test; ^3^ Cambridge Neuropsychological Test Automated Battery (CANTAB)	TMT: 0 DSST: 0 Stroop test: 0 CANTAB: 0	Some concerns
O’Toole et al., 2025 [26]	Cognition: ^4^ Computerized cognitive test battery	Computerized cognitive battery: 0	Some concerns
Tharoor et al., 2023 [27]	Cognition: ^5^ National Institute for Mental Health and Neurosciences (NIMHANS) Neuropsychological battery	NIMHANS Neuropsychological battery: 0	Some concerns
**L-carnosine with other nutrients or bioactive compounds**			
Cornelli et al., 2010 [19]	Cognition: MMSE	MMSE: ++	Low risk
Ding et al., 2018 [10]	Cognition: ADAS-Cog; MMSE; WMS-LM Mood and depression: BDI Quality of Life: SF-36 Brain structure and function: MRI, MRI–Diffusion Tensor Imaging, pASL (PFC)	ADAS-Cog: 0 MMSE: 0 WMS-LM1: ++; WMS-LM2: ++ BDI: 0 SF-36: 0 MRI-Diffusion Tensor image: ++ pASL (PFC): ++	Some concerns
^6^ Hisatsune et al., 2016 [16]	Cognition: ADAS-Cog; WMS-LM Mood and depression: BDI Quality of Life: SF-36 Brain structure and function: pASL (PCC)	ADAS-Cog: 0 WMS-LM1: 0; WMS-LM2: ++ BDI: 0 SF-36: 0 pASL (PCC): ++	Some concerns
^6^ Katakura et al., 2017 [17]	Cognition: ADAS-Cog; WMS-LM Mood and depression: BDI Quality of Life: SF-36	ADAS-Cog: 0 WMS-LM1: 0; WMS-LM2: + BDI: 0 SF-36: 0	Some concerns
^6^ Rokicki et al., 2015 [18]	Cognition: ADAS-Cog; WMS-LM Mood and depression: BDI Brain structure and function: rsfMRI (DMN, RFPN, and PCC)	ADAS-Cog: 0 WMS-LM1: 0; WMS-LM2: ++ BDI: 0 rsfMRI (DMN, RFPN, and PCC): 0/++	Some concerns
Masuoka et al., 2019 [20]	Cognition: ADAS-Cog; CDR; MMSE; WMS-LM Mood and depression: GDS Brain structure and function: EEG	ADAS-Cog: 0 global CDR: ++; CDR sum of boxes: 0 MMSE: 0 WMS-LM1: 0; WMS-LM2: 0 GDS: 0 sNAT (EEG spectrum intensity): +	Some concerns
Shirotsuki et al., 2017 [21]	Mood and depression: POMS	POMS-TA: + POMS-D: 0 POMS-F: ++	High risk
Szcześniak et al., 2014 [22]	Cognition: MMSE; STMS Mood and depression: GDS	MMSE: 0 STMS: 0 GDS: 0	High risk

ADAS-Cog, Alzheimer’s Disease Assessment Scale–Cognitive Subscale; BDI, Beck Depression Inventory; CDSS, Calgary Depression Scale for Schizophrenia; CDR, Clinical Dementia Rating; CPT, Continuous Performance Test; DMN, default mode network; EEG, Electroencephalogram; GDS, Geriatric Depression Scale; HAM-D, Hamilton Depression Rating Scale; MMSE, Mini–Mental State Examination; MRI, Magnetic Resonance Imaging; pASL, 3D pulsed arterial spin labeling; PCC, posterior cingulate cortex; PFC, prefrontal cortex; POMS, Profile of Mood States; POMS-TA, POMS Tension-Anxiety; POMS-D, POMS Depression; POMS-F, POMS Fatigue; QOLI, Quality of Life Interview; RFPN, right fronto-parietal network; ROB, risk of bias; rsfMRI, Resting-state functional Magnetic Resonance Imaging; SF-36, 36-Item Short Form Health Survey; SST, Set Shifting test; STDT, Strategic Target Detection; STMS, Short Test of Mental Status; TMT, Trail Making Test; WAIS-R Digit Symbol, WAIS-R Digit Symbol Substitution Test; WMS-LM, Wechsler Memory Scale (WMS)–Logical Memory. ^1^ Key findings compare carnosine intervention to placebo groups unless otherwise noted: ++ significant beneficial effects (*p* < 0.05); + marginally significant beneficial effects (0.05 < *p* <0.1); 0 no effects. ^2^ Overall ROB is defined as high risk, some concern, or low risk based on the Cochrane risk-of-bias tool for randomized trials (RoB-2). ^3^ Cambridge Neuropsychological Test Automated Battery: Delayed Match to Sample (DMS), Paired Associates Learning (PAL), Pattern Recognition Memory (PRM), Reaction Time Index (RTI), Rapid Visual Processing (RVP), Spatial Working Memory (SWM). ^4^ Computerized cognitive test battery: Motor Praxis (MP), Visual Object Learning (VOLT), Fractal 2-back (F-2B), Abstract Matching (AM), Line Orientation (LOT), Emotion Recognition (ERT), Matrix Reasoning (MRT), Digit Symbol Substitution (DSST), Balloon Analog Risk (BART), Psychomotor Vigilance (PVT). ^5^ National Institute for Mental Health and Neurosciences (NIMHANS) Neuropsychological battery: TMT, Digit Vigilance Test, Auditory Verbal Learning Test (AVLT), The Tower of London. ^6^ Participants in these articles originated from the same study conducted within the same recreated period.

**Table 4:** Characteristics, key findings, and overall risk of bias of single-arm clinical trials.

Author, Year (Reference)	Country	Funding Source	N	Design	Mean Age [Range], Years ^1^	% Male	Health Status	Carnosine Intervention and Dosage	Intervention Duration	Outcome Analyzed	Key Findings ^2^	Overall ROB ^3^
Sakae et al., 2020 [29]	Japan	None	29	Single-arm study	~36.0 [NR]	28%	Patient with binge eating disorder or bulimia nervosa	Zinc–L-carnosine 75 mg/d for 4 weeks, and 150 mg/d for 12 weeks	16 weeks	Mood and depression: QIDS-SR16	QIDS-SR16: ++	High risk

QIDS-SR16, Quick Inventory of Depressive Symptomatology; ROB, risk of bias. ^1^ If the total mean (SD) was not reported for study participants, the table shows means as weighted averages (indicated by the “~” symbol) and includes separate group SDs reported in the original study (presented in parentheses, separated by a comma). ^2^ Key findings compare carnosine intervention to placebo groups unless otherwise noted: ++ significant beneficial effects (*p* < 0.05). ^3^ Rated as high risk of bias due to no control group.

## Data Availability

The original contributions presented in the study are included in the article, further inquiries can be directed to the corresponding author.

## Supplementary Materials

The following supporting information can be downloaded at: https://www.mdpi.com/article/10.3390/nu18091385/s1, Supplementary Material S1: Search strategy of scoping review; Supplementary Material S2: Overview of Carnosine Scoping Review; Table S1: Study eligibility criteria considered for scoping review; Table S2: GRADE evidence profiles for outcomes assessed by included studies; Figure S1: Risk of bias assessment using the Cochrane Risk of Bias Tool 2.0 for 12 included randomized controlled trials; Figure S2: Sensitivity analysis using alternative r values (i.e., 0.5) to impute missing SD for WMS-LM1 and WMS-LM2. Figure S3: Sensitivity analysis using alternative r values (i.e., 0.5) to impute missing SD for MMSE. Box size indicates study weight. WMS-LM1 = Wechsler Memory Scale–Local Memory Immediate Recall; WMS-LM2 = Wechsler Memory Scale–Local Memory Delay Recall.

## Abbreviations

The following abbreviations are used in this manuscript:

BDI	Beck Depression Inventory
CBT	Cognitive Behavioral Therapy
CDR	Clinical Dementia Rating
CI	Confidence Interval
EEG	Electroencephalogram
GDS	Geriatric Depression Scale
MMSE	Mini–Mental State Examination
MRI	Magnetic Resonance Imaging
POMS	Profile of Mood States
PI/ECO	Population Intervention/Exposure Comparator Outcome
PRISMA	Preferred Reporting Items for Systematic Reviews and Meta-Analyses
QOL	Quality Of Life
RCT	Randomized Controlled Trial
ROB	Risk of Bias
RoB 2	Cochrane Risk-of-Bias Tool for Randomized Trials
SD	Standard Deviation
SF-36	36-Item Short Form Health Survey
SR	Systematic Review
STMS	Short Test of Mental Status
WMS	Wechsler Memory Scale
WMS-LM1	WMS–Local Memory Immediate Recall
WMS-LM2	WMS–Local Memory Delayed Recall
