# Supplementary material for: The Effects of Carnosine on Cognitive Function and Mental Health—A Systematic Review and Meta-Analysis
Source: Nutrients. 2026 Apr 28;18(9):1385. doi: 10.3390/nu18091385 (PMC13165363; doi:10.3390/nu18091385)
Supplement: Supplementary file 1 [file nutrients-18-01385-s001.zip › nutrients-4244325-supplementary.pdf]

Supplementary Material S1: Search strategy of scoping review

1. Pubmed search strategy:

(("carnosine"[Supplementary Concept] OR "carnosine"[All Fields] OR "carnosine"[MeSH Terms] OR "carnosine s"[All Fields]) OR "beta-alanyl-L-histidine"[All Fields] OR "beta alanyl l histidine"[All Fields] AND ((2006/1/1:2025/6/26[pdat]) AND (chinese[Filter] OR english[Filter]))) NOT (("carnosine"[Supplementary Concept] OR "carnosine"[All Fields] OR "carnosine"[MeSH Terms] OR "carnosine s"[All Fields]) OR "beta-alanyl-L-histidine"[All Fields] OR "beta alanyl l histidine"[All Fields] AND ((meta-analysis[Filter] OR review[Filter] OR scopingreview[Filter] OR systematicreview[Filter]) AND (2006/1/1:2025/6/26[pdat]) AND (english[Filter])))

2. Embase search strategy:

('carnosine'/exp OR carnosine:ti,ab,kw OR 'beta-alanyl-l-histidine':ti,ab,kw)

AND [2006-2025]/py NOT [medline]/lim

3.

4. Cochrane Central search strategy:

   Key word: carnosine OR "beta-alanyl-l-histidine."

   Publication Year: 2006 to 2025

   Language: English

Supplementary Material S2: Overview of Carnosine Scoping Review

Supplementary Table S1. Study eligibility criteria considered for scoping review.

Category	Inclusion criteria	Exclusion criteria
Study design	• Any intervention study:<br>• Randomized controlled trials<br>• Non-randomized controlled trials<br>• Randomized or non-randomized cross-over trials<br>• Single-arm trials	• Case-control studies<br>• Cohort studies<br>• Cross-sectional studies<br>• Narrative reviews<br>• Systematic reviews and meta-analysis<br>• Protocols<br>• Conference proceedings<br>• Abstracts<br>• Letters to the editor<br>• Case studies or case series<br>• In vitro and animal studies
Populations	Human subjects without age, sex, and disease restrictions	Not human subjects
Intervention or exposure	Oral intake of L-carnosine (β-alanyl-L-histidine) or Zinc-L-carnosine alone or combined with other supplements, including food extract with carnosine (ex: chicken essence or chicken broth extract)	Interventions are not administered orally (for example, eyedrops, skin cream or topical products).
Comparators	Placebo or other supplements	None
Outcomes	* Examples below are included, but not limited to*<br>1. **Muscle function**<br>For example, muscle strength, power, endurance, exercise performance, fatigue resistance, and physical performance (ex: sit to stand function)	Outcomes that are not related to sarcopenia, muscle function, brain and cognitive function, wound healing, pain management, energy metabolism (ATP & mitochondria), or cancer treatment.<br><br>Chronic diseases not listed in the inclusion criteria (e.g., CKD,

	2. **Brain and cognitive function**<br>For example, global cognitive function assessment, brain image markers, behavior assessment, and psychosocial assessment (ex: mood, depression, and quality of life)<br>3. **Wound healing**<br>For example, wound healing rate, wound appearance, wound-related biochemical markers, and visual function recovery<br>4. **Pain management**<br>For example, pain levels and use of painkillers<br>5. **Energy metabolism (ATP & mitochondria):**<br>For example, glycolysis and pH regulation, anti-glycating activity, and mitochondrial protection and function<br>6. **Chronic disease management**<br>Obesity and Overweight: (ex: blood glucose control, blood lipid profile; weight management and reduction)<br>Diabetes management (ex: blood sugar and HbA1C control)<br>Cardiovascular disease control (ex: cholesterol control)<br>Heart function (ex: heart failure recovery)	hemodialysis, HCV, etc.)<br><br>ADHD, autism, and obsessive-compulsive disorder (OCD), as well as other mental illnesses, psychiatric disorders, or neurodevelopmental disorders not listed in the inclusion criteria.

	7. **Cancer treatment**<br>For example, inhibiting or attenuating tumor growth, increasing the survival rate, has potential as a complementary therapy, and reducing side effects from chemotherapy drugs.	
Date of publication	From 2006 to present	Before 2006
Publication status	Articles published in peer-reviewed journals	Articles not published in peer-reviewed journals, including unpublished data, manuscript reports, abstracts, pre-prints, and conference proceedings
Study duration	No restriction	N/A
Sample size	No restriction	N/A
Language of publication	English, Chinese	Languages other than English and Chinese

Study Selection

Following the PRISMA guidelines, the identification process started with importing 2809 records from databases such as Cochrane Central, Embase, and PubMed. Initially, 124 records were removed due to duplicates and supplemental records, leaving 2685 records for abstract screening. During this phase, 2517 records were excluded after dual review with the principal reviewer, resulting in 168 reports for retrieval. In the full-text review stage, 95 reports were excluded for reasons including incorrect study design, incorrect intervention, incorrect outcome, incorrect publication type, and other reasons. Ultimately, 73 reports were included in the final review. This systematic process ensured a thorough evaluation of the available literature, adhering

to the PRISMA guidelines.

Brief Results of Data Extraction

This scoping review identified and analyzed 73 relevant articles investigating the health outcomes of carnosine and anserine supplementation. The most common studies used L-carnosine, accounting for 44 articles. Specific chelated forms, such as zinc carnosine, comprised an additional 18 studies. Moreover, supplements derived from food extracts, like chicken essence or chicken broth extract, were examined in 11 articles. Studies involving only carnosine supplements totaled 34 articles, while an additional 39 studies investigated carnosine in combination with other ingredients.

Regarding health outcomes, these articles mainly focused on two areas: brain and cognitive function (21 articles) and chronic disease management (20 articles). Other important outcomes included sarcopenia and muscle function (13 articles), cancer management (11 articles), and pain management (9 articles). Less-studied areas were wound healing (8 articles) and energy metabolism (2 articles). These findings demonstrate a strong research interest in the neurological and systemic health benefits of these supplements.

Supplementary Table S2. GRADE evidence profiles for outcomes assessed by included studies.

**Quality assessment**							**Summary of findings**	**Strength of Evidence** [1]
**No of studies**	**Design**	**Limitations**	**Inconsistency**	**Indirectness**	**Imprecision**	**Publication bias**		
**Cognitive function outcomes**								
10	10 RCTs	**Some concerns:**<br>Among 10 RCTs, 2 reported low ROB, 7 had some concerns, and 1 had high ROB.	**No serious inconsistency:**<br>In 5 RCTs consistently showed no significant difference in most cognitive outcomes between L-carnosine and control.<br>In another 5 RCTs, 3 showed a significant difference in WMS-LM2 score (I^2 = 58.3%), and 4 showed improvement in MMSE (I^2 = 21.3%) after L-carnosine supplementation combined with anserine or other antioxidants compared to placebo. No significant effect on WMS-LM1 (I^2 = 8.5%) and other cognitive outcomes.	**No serious indirectness:**<br>Clinical outcome.	**Serious Imprecision:**<br>9 RCTs had small sample size (n <=100), and only 1 RCT had a appropriate sample size (n=299).	**Suspected publication bias**:<br>Some RCTs didn't report their protocol.	Five RCTs evaluated L-carnosine supplementation alone versus placebo and mostly found no significant differences in cognitive function measures between L-carnosine and placebo, except that 2 RCTs reported improvements on the WAIS-R Digit Symbol, SST, and STDT with L-carnosine supplementation compared to placebo.<br><br>Another 5 RCTs compared L-carnosine supplementation combined with anserine or other antioxidants to placebo. Among these, 3 RCTs showed improvements in WMS-LM2 scores (pooled net change = 1.70; 95% CI 0.19, 3.2) but not in WMS- LM1 scores (pooled net change = 0.76; 95% CI -0.18, 1.71) compared to placebo. 4 of the 5 RCTs showed that MMSE scores were significantly improved compared to placebo (pooled net change = 0.62; 95% CI 0.23, 1.01).<br><br>1 RCT found that daily 1 g of anserine/L-carnosine (3:1) produced a significantly greater change in global CDR than placebo, while another 1 RCT showed no effect on the STMS after daily 1 g of anserine/carnosine (2:1).	⊕⊕OO<br>LOW[2]
**Brain structure and function outcomes**								
3	3 RCTs	**Some concerns:**<br><br>All studies have some concerns for overall ROB.	**No serious inconsistency:**<br>All trials reported marginally or significant changes in brain structure-related outcomes.	**Serious indirectness:**<br>Brain structure measurements are used to support cognitive function outcomes. They are indirect evidence.	**Serious Imprecision:**<br>All study have small sample sizes.	**Suspected publication bias**:<br>Only 1 study reported protocol information. The sample sizes in all studies are also small.	pASL:<br>2 RCTs showed significant preservation of cerebral blood flow in anserine/carnosine supplementation. Both RCTs reported increased perfusion in the PCC region ($p < 0.001$ in Ding et al., 2018, $p < 0.01$ in Hisatsune et al., 2016), but this significance was not observed with inverse calculation.	⊕OOO<br>VERY LOW[3]

							Diffusion MRI:<br>1 RCT reported attenuated decline in white matter integrity (fractional anisotropy) in the intervention group (p = 0.003).<br><br>rsfMRI:<br>1 RCT found significant changes in intervention group's functional connectivity within RFPN, PCC, and DMN masks (p < 0.05) in comparison of control group.<br><br>EEG:<br>1 RCT showed the trend of improvement in EGG results in carnosine group, but the overall effect was not significant between groups.	
**Mood and depression outcomes**								
10	9 RCTs and 1 single-arm study	**Some concerns**: All trials have some or high ROB in at least 1 ROB domain. For each ROB domain, except for measurement of outcome, all of the trials have low ROB	**No serious inconsistency:**<br>In 7 RCTs consistently show no significant association.<br>One RCT found a significant improvement in HAM-D.<br>One RCT found significantly improved POMS Tension-Anxiety and Fatigue scores.<br>One single-armed study found steadily decrease in QIDS-SR16.	**No serious indirectness:**<br>Clinical outcomes.	**Serious Imprecision**<br>All studies have small sample sizes. The one study that reports significantly greater improvement in HAM-D has large CI (0.45–5.84).	**Suspected publication bias**:<br>Some RCTs didn't report their protocol.	Mood and depression outcomes were mixed across 7 RCTs and 1 single-arm trial. One RCT showed improved depression scores with L-carnosine alone, while most studies found no significant differences; one study improved anxiety and fatigue, and the single-arm trial showed reduced depressive symptoms.	⊕⊕OO<br>LOW[4]
**Quality of life (QOL) outcomes**								
4	4 RCTs	**Some concerns**:<br>Only 1 article has low-risk overall ROB. The other 5 articles have some concerns in overall ROB.	**No serious inconsistency:**<br>All trials reported no significant changes in SF-36 between carnosine	**Serious indirectness:**<br>Quality of life is a secondary outcome in all articles.	**Serious Imprecision**<br>All study have small sample sizes.	**Suspected publication bias**:<br>Only 2 studies provided protocol information.	All RCTs show no significant difference between groups.	⊕OOO<br>VERY LOW[5]

			intervention group and control group.					

CDR, Clinical Dementia Rating; EEG, Electroencephalogram; GRADE, Grades of Recommendation, Assessment, Development, and Evaluation; HAM-D, Hamilton Depression Rating Scale; MMSE, Mini-Mental State Examination; MRI, Magnetic Resonance Imaging; pASL, pulsed arterial spin labeling; PCC, Posterior Cingulate Cortex; POMS, Profile of Mood States; QIDS-SR16, Quick Inventory of Depressive Symptomatology; QOL, Quality of Life; RFPN, Right Frontoparietal Network; ROB, risk of bias; rsfMRI, Resting-state functional Magnetic Resonance Imaging; RCTs, Randomized Controlled Trials; SF-36, The 36-Item Short Form Health Survey; SST, Set Shifting test; STDT, Strategic Target Detection; STMS, Short Test of Mental Status; WAIS-R Digit Symbol, WAIS-R Digit Symbol Substitution Test; WMS-LM1, Wechsler Memory Scale (WMS)-Logical Memory; WMS-LM2, Wechsler Memory Scale (WMS)-Logical Memory; WISC-Digit Span, Wechsler Intelligence Scale for Children (WISC)-Digit Span.

[1] Symbols indicate the following strength of evidence: ⊕⊕⊕⊕High (We are very confident that the true effect lies close to that of the estimate of the effect.); ⊕⊕⊕◯, Moderate (We are moderately confident in the effect estimate: The true effect is likely to be close to the estimate of the effect, but there is a possibility that it is substantially different.); ⊕⊕◯◯, Low (Our confidence in the effect estimate is limited: The true effect may be substantially different from the estimate of the effect.); and ⊕◯◯◯, Very low (We have very little confidence in the effect estimate: The true effect is likely to be substantially different from the estimate of effect).

[2] The certainty of evidence for cognitive outcomes was rated as low due to risk of bias, imprecision, and suspected publication bias, suggesting limited confidence in the current evidence and the need for further high-quality studies.

[3] The certainty of evidence for brain structure and function outcomes, used as indirect measures of cognitive function, was rated as very low. In addition to risk of bias, imprecision, and suspected publication bias, these outcomes were further downgraded for indirectness, indicating very limited confidence in the findings.

[4] The certainty of evidence for mood and depression outcomes was rated as low due to risk of bias, imprecision, and suspected publication bias. Most studies found no significant association, although two RCT and one single-arm trial reported improved depressive symptoms, suggesting the true effect may differ and further high-quality studies are needed.

[5] The certainty of evidence for quality of life outcomes was rated as very low, due to risk of bias, indirectness, imprecision, and suspected publication bias. The current evidence remains limited and further well-designed studies are needed to confirm these findings.

Supplemental Figure S1. Risk of bias assessment using the Cochrane Risk of Bias Tool 2.0 for 12 included randomized controlled trials.

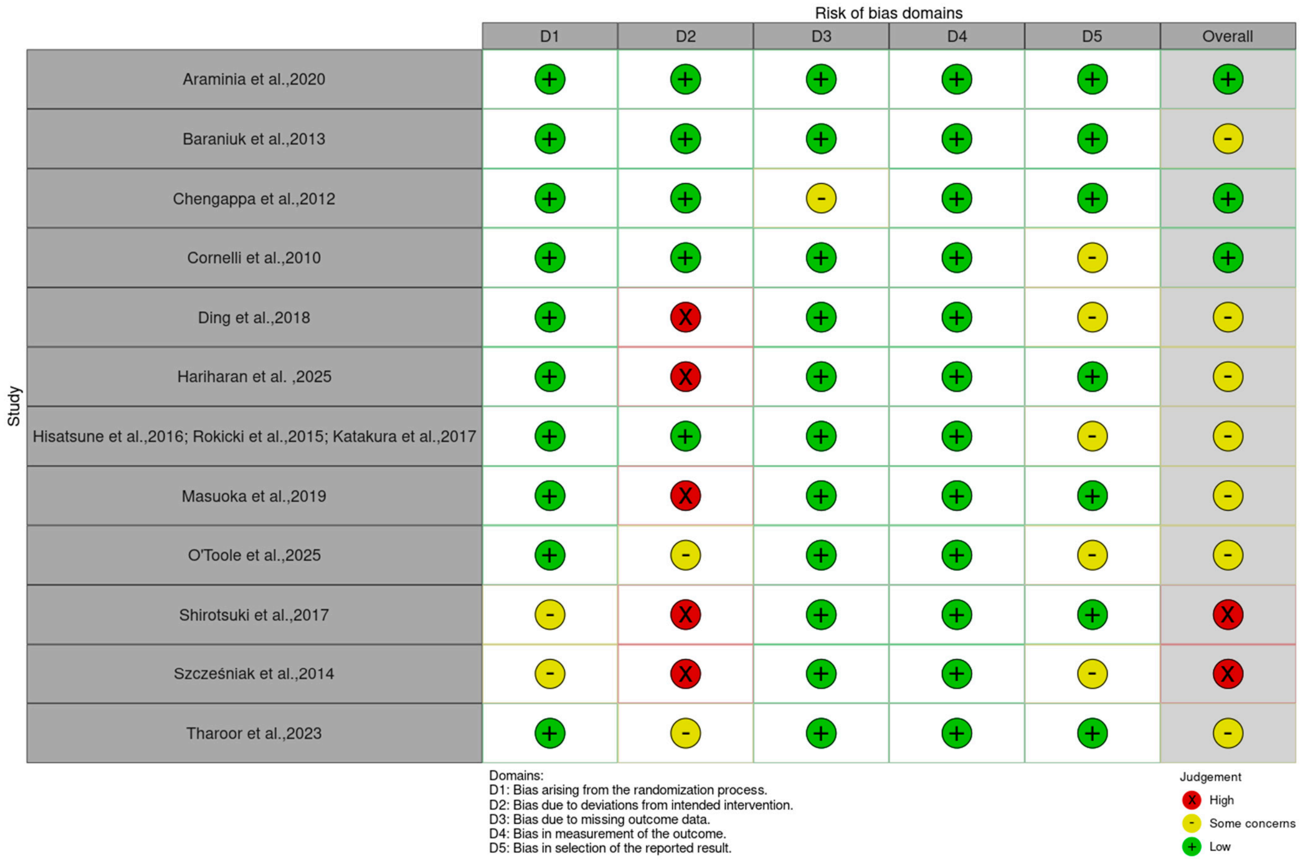

Supplemental Figure S2. Sensitivity analysis using alternative r values (i.e., 0.5) to impute missing SD for WMS-LM1 and WMS-LM2. Random-effect meta-analysis of randomized controlled trials assessing WMS-LM1 and WMS-LM2 outcomes comparing supplementation with anserine and carnosine (3:1) to placebo. Box size indicates study weight. WMS-LM1 = Wechsler Memory Scale – Local Memory Immidate Recall; WMS-LM2 = Wechsler Memory Scale – Local Memory Delay Recall.

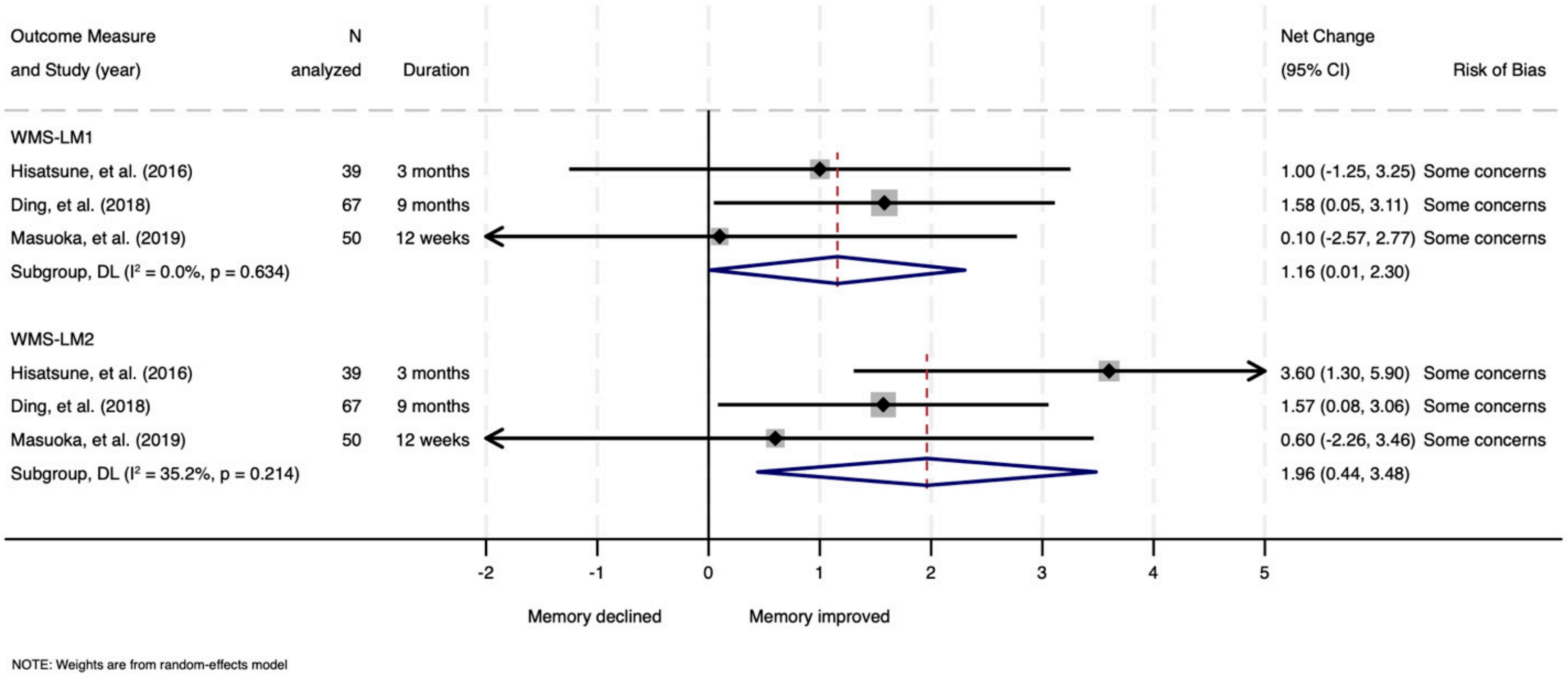

Supplemental Figure S3. Sensitivity analysis using alternative r values (i.e., 0.5) to impute missing SD for MMSE. Random-effects model meta-analysis of randomized controlled trials measuring MMSE in participants given dietary supplements containing carnosine. Box sizes represent study weight. MMSE, Mini-Mental State Examination.

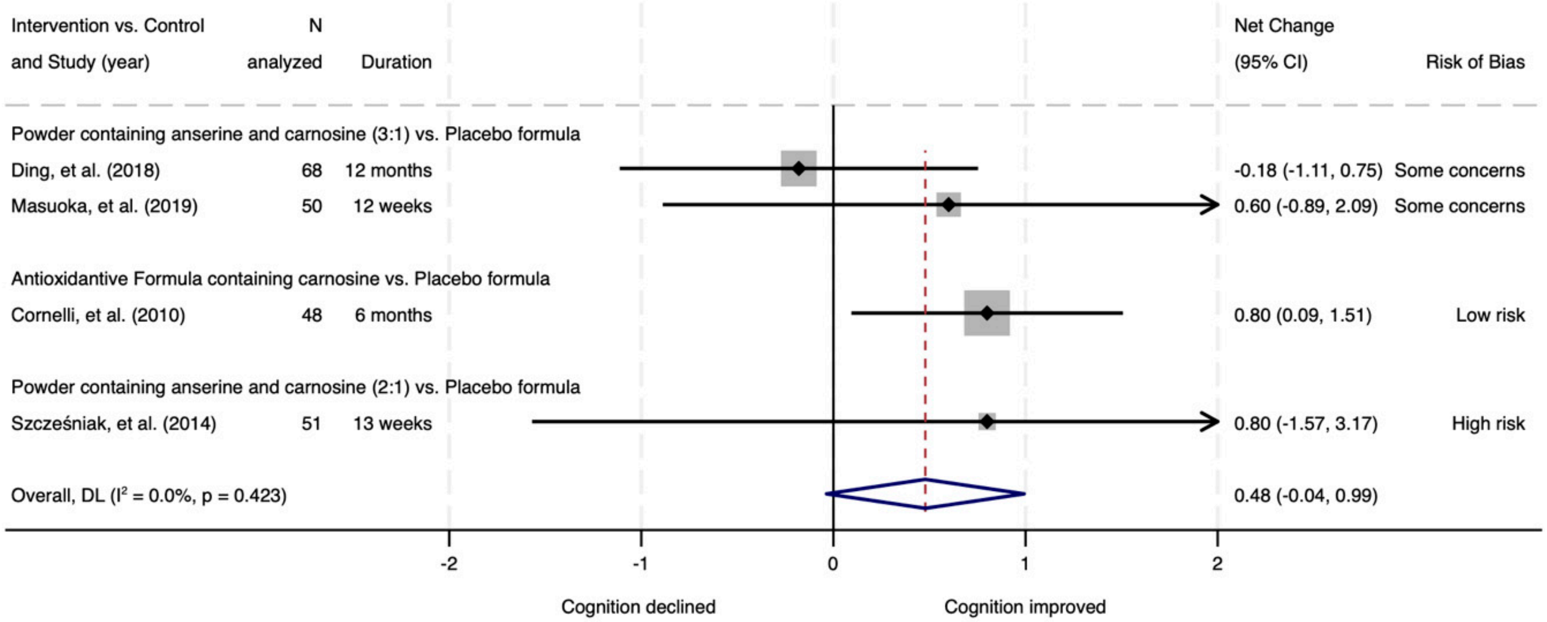

NOTE: Weights are from random-effects model
